# Reduced Flavin: NMR investigation of N(5)-H exchange mechanism, estimation of ionisation constants and assessment of properties as biological catalyst

**DOI:** 10.1186/1471-2091-6-26

**Published:** 2005-11-25

**Authors:** Peter Macheroux, Sandro Ghisla, Christoph Sanner, Heinz Rüterjans, Franz Müller

**Affiliations:** 1 Graz University of Technology, Institute of Biochemistry, Petersgasse 12, A-8010 Graz, Austria; 2 Fachbereich Biologie, Universität Konstanz, D-78457 Konstanz, Germany; 3 Institut fur Biophysikalische Chemie, J.W. Goethe-Universität, Biozentrum N230, Marie-Curie-Strasse 9, D-60439 Frankfurt am Main, Germany; 4 Wylstrasse 13, CH-6052 Hergiswil, Switzerland

## Abstract

**Background:**

The flavin in its FMN and FAD forms is a versatile cofactor that is involved in catalysis of most disparate types of biological reactions. These include redox reactions such as dehydrogenations, activation of dioxygen, electron transfer, bioluminescence, blue light reception, photobiochemistry (as in photolyases), redox signaling etc. Recently, hitherto unrecognized types of biological reactions have been uncovered that do not involve redox shuffles, and might involve the reduced form of the flavin as a catalyst. The present work addresses properties of reduced flavin relevant in this context.

**Results:**

N(5)-H exchange reactions of the flavin reduced form and its pH dependence were studied using the ^15^N-NMR-signals of ^15^N-enriched, reduced flavin in the pH range from 5 to 12. The chemical shifts of the N(3) and N(5) resonances are not affected to a relevant extent in this pH range. This contrasts with the multiplicity of the N(5)-resonance, which strongly depends on pH. It is a doublet between pH 8.45 and 10.25 that coalesces into a singlet at lower and higher pH values. From the line width of the ^15^N(5) signal the pH-dependent rate of hydrogen exchange was deduced. The multiplicity of the ^15^N(5) signal and the proton exchange rates are little dependent on the buffer system used.

**Conclusion:**

The exchange rates allow an estimation of the pK_a _value of N(5)-H deprotonation in reduced flavin to be ≥ 20. This value imposes specific constraints for mechanisms of flavoprotein catalysis based on this process. On the other hand the pK ≈ 4 for N(5)-H protonation (to form N(5)^+^-H_2_) would be consistent with a role of N(5)-H as a base.

## Background

The isoalloxazine ring system is the redox active moiety of the coenzyme forms (FMN or FAD) present in flavoenzymes. These are involved in a variety of biological processes, spanning a wide spectrum with regard to the underlying chemical reaction mechanisms. These range from the classical (de)hydrogenation, the uptake, release and transport of electrons, the production of light (bioluminescence), photochemistry of the reduced form (as in photolyases), light signal transduction (as in blue light receptors), activation of oxygen and redox sensing, to name only the most prominent ones [1]. In addition to these functions, others have emerged that appear to require hitherto unrecognized roles of reduced flavin in chemical catalysis, such as reactions that are redox-neutral (for a review see [2]). These will be addressed briefly below.

In reduced flavin, N(5) is crucial for the functioning of the isoalloxazine system as it is the locus involved in uptake/release of redox equivalents and is in general in contact with reacting ligands. In the intermediate pH range reduced flavin N(5) exists in its neutral, N(5)-H form, it can be protonated to yield N(5)H_2_^+ ^at low pH and might exist in the anionic form N(5)^- ^at very high pH (*cf *Fig. 5, below). Based on kinetic arguments [3] Bruice and co-workers have estimated the pK_a _for deprotonation of this group as being around 20 in free flavin. Urban and Lederer, on the other hand, imply a value around 15 for flavocytochrome b_2 _[4] and it has been postulated that this pK might even be lower than 7 [5]. A method useful for the assessment of the properties of reduced flavin, and specifically of N(5) is the nuclear magnetic resonance (NMR) spectroscopy. It has been used to study the interactions of apoprotein and flavin, and the perturbations of these interactions induced by binding of substrate/ligands[6]. In these studies, we have observed that the N(5)H group in most two-electron reduced flavoproteins appears as a doublet in the ^15^N-NMR spectrum due to the N(5)-H coupling, while free flavin exhibits a singlet in the pH range 5–8 due to fast proton exchange. Therefore, the doublets observed in reduced flavoproteins have been interpreted as resulting from the absence or from slow proton exchange on the NMR time scale. This can result from inaccessibility of the N(5)H group to bulk water. However, no systematic study on the basic mode of N(5)-H exchange in free reduced flavin in aqueous solution is available. The great variety of chemical reactions mentioned above raises the question about the physical interactions between the apoprotein and the coenzyme that are responsible for the tuning necessary to catalyze particular reactions.

A detailed knowledge of this process and of its mechanism would provide several insights into flavoenzyme structure and function: a) It would provide a basis for the interpretation of ^15^N-NMR spectra of reduced flavoproteins. b) It could help understand a variety of exchange processes of substrate/product-linked hydrogens in various dehydrogenation reactions involving flavoproteins. c) It might help clarify the possible role of reduced flavin in the β-elimination of halide from β-halogenated substrates catalyzed by several flavoproteins. X-ray structural information has shown that there are no basic amino acid residues at the active centers of e.g. (oxidized) D-amino acid oxidase [7-9]. The hypothesis has thus been put forward that N(5) of the reduced enzyme flavin is the base that is involved in enzyme catalyzed elimination [10]. In addition labeled hydrogen is abstracted from the α position and is partially incorporated into the β position of product [11]. These data would also be compatible with the proposed role of reduced flavin N(5)-H being involved in label transposition/exchange [7-9,12]. d) Chorismate synthase catalyzes a redox-neutral anti-1,4-elimination of a hydrogen and a phosphate group and has an absolute requirement for a reduced FMN cofactor. Again, it has been assumed that an amino acid functional group serves as a base in the elimination of the hydrogen but the recent elucidation of the 3D-structure has revealed that the only functional group that could be invoked in this process is the N(5) position of the flavin [12,13]. e) Based on recent studies it has been proposed that in the dehydrogenation reaction catalyzed by monoamine oxidase, the flavin N(5) of the postulated C(4a)-flavin substrate adduct (which is isoelectronic with the flavin hydroquinone) acts as a base in hydrogen abstraction [14]. Thus, a critical functional role of the flavin N(5) nitrogen in its reduced 1,5-dihydro, or its isoelectronic 4a,5-dihydro forms appears to emerge. The present study was undertaken to increase our understanding of processes involving this position and specifically the pH-dependent proton exchange and to gain information on the basicity/nucleophilicity of N(5) in reduced flavin, which provides basic information on possible mechanistic roles in flavoenzyme catalysis.

## Results and Discussion

### Chemical shifts

The pH-dependence of the ^15^N chemical shifts of reduced free flavin (N(l), N(3), N(5), N(10)) has been previously investigated at pH 5.2 – 8 [15], a pH range where most flavoproteins are active and stable. This study revealed that the chemical shift of the N(1) atom in reduced free flavin is strongly pH-dependent due to its ionization. A pK_a _value of 6.8 was calculated for this process [15]. Also the signal of the N(10) atom shows a similar pH-dependence; the chemical shift difference between that of the neutral and of the anionic species, however, is smaller than that of the N(1) atom. This observation was rationalized previously as resulting from a change of sp^2 ^hybridization of the N(10) atom [15,16]. The chemical shifts of the N(3) and the N(5) atom are practically independent of pH in the range studied. In aqueous solution only a singlet was observed for the N(5)H and the N(3)H groups. The signal of the N(3)H group remains as a sharp, narrow line over the pH range studied, whereas that of the N(5)H group exhibits a sharp line at pH 5.4 and a broadened one at pH 7 and higher [15,16].

In the present study the pH-dependence of the ^15^N chemical shifts of reduced free flavin was extended to the pH range 4.0 to 12.3. The ^15^N chemical shifts at pH 4.0 are identical, or almost identical to those observed earlier at pH 5.0 [15,16], with the exception that the chemical shift of the N(1) atom is shifted upfield by 1.4 ppm. Also at pH 12.3 the ^15^N chemical shifts of the N(5) and N(10) atoms are identical with those reported previously for pH 8.0 [15,16]. The ^15^N chemical shift of the N(3) atom shows a slight downfield shift (+1.9 ppm) at pH >11 indicating the onset of deprotonation of the N(3)H group. Also a small downfield shift (+0.7 ppm) is observed for the N(1) atom in the high pH region, probably related to the (partial) ionization of the N(3)H group.

### Signal structure of the N(5)-H resonance

**Figure 1:**
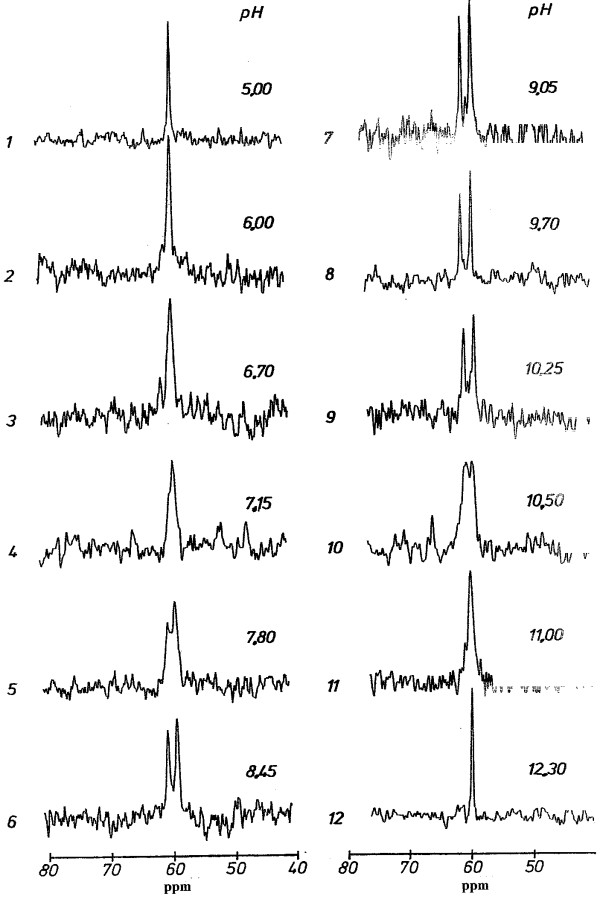
**Multiplicity of the ^15^N-NMR signal of the reduced FMN N(5)H group as a function of pH**. The flavin concentration was 5 mM in 250 mM Tris + 100 mM NaCl. The obtained FID was processed further by exponential multiplication using a line broadening factor of 20 Hz in order to improve the signal to noise ratio.

The two nitrogen atoms in reduced free flavin bearing a hydrogen are N(3) and N(5). The signal of N(3) appears as a singlet in the pH range studied and from this rapid exchange can be assumed. As shown in Fig. 1 the shape of the N(5) resonance signal depends strongly on the pH. At low pH values there is a sharp singlet that broadens with increasing pH. Above pH 7.15 the broad singlet begins to show a doublet structure (first coalescence point), the resolution of which increases and finally gives rise to a doublet (pH 8.45 – 10.25). Further increase of the pH leads to broadening of the doublet lines again and eventually to a second coalescence point around pH 10.5 (see also below). At even higher pH values the signal becomes again a sharp singlet. These data demonstrate that below the first and above the second coalescence point we have two pH regions of fast hydrogen exchange. This is supported by the fact that the line widths of the decoupled and undecoupled resonance lines are identical (4 Hz, natural line width) at pH 12.3 and pH 5.0. In the pH range between the two coalescence points the exchange is comparatively slow. The asymmetric shape of the doublet (higher intensity of the high field line in Fig. 1, pH 8.45 – 10.25) can be a result of either the high magnetic field (11.4 Tesla), which causes a prevailing contribution of the CSA-relaxation mechanism [17] or could be due to a particular proton exchange mechanism [18]. Which of the two effects is prevailing, cannot be deduced from the present data.

The ^1^J^15 ^N(5)-H coupling constant can be determined from the spectra obtained in the slow exchange region (Fig. 1, spectra 7 and 8) and was found to be 85 Hz. This value agrees well with that determined previously in a CHCl_3 _solution (87.5 Hz) [15]. The coupling constant of reduced flavin is similar to those reported for eneamines and aniline derivatives [19]. ^1^J^15^N-H coupling constants are mainly governed by the hybridization of the nitrogen atom. A low s-character gives rise to small coupling constant (the coupling constant for tetrahedral ammonia is 73 Hz) whereas a high s-character results in a high coupling constant (for linear nitriles coupling constants as high as 135 Hz have been found [20]). The observed coupling constant of 85 Hz, which is similar to the value found for pyrrole (96.5 Hz [20]), indicates that the N(5) atom possesses approximately 31 % s-character, i.e. it is highly sp^2 ^hybridized. The pH-dependence of the shape of the N(5)-resonance was not affected by buffer systems like Tris, phosphate and borate.

### Determination of N(5)-Hydrogen exchange rates

The exchange rate of the N(5) hydrogen was determined using equation (1) for fast exchange (below pH 7.8 and above pH 10.5) [21] and equation (2) for slow exchange (between pH 7.8 and 10.5) [22]:

k = 1/τ_e _= 4 π pA pB Δν^2^/(Δν_1/2 _- Δν_1/2_°)     (1)

k = 1/τ_e _= π (Δν_1/2 _- Δν_1/2_°)     (2)

with k as the exchange rate; τ_e _the lifetime of each state, Δν_1/2 _the half line width of the resonance line, Δν_1/2_° the half line width of the proton decoupled signal which should be equal to the so-called natural line width (4 Hz, see above) under conditions of fast proton exchange. Δν is the difference of the resonance frequencies of the exchanging species under the condition of slow exchange, i.e. equal to the coupling constant ^1^J_N-H_, pA and pB are the molar fractions of the exchanging species in state A and B, respectively. The exchange rates have also been determined in phosphate and borate buffers at a few selected pH values; they are in the range of those determined in Tris buffer. The signal structure at pH 10.5 (transition from doublet to singlet) indicates that the coalescence point is close to this pH value. This is supported by the fact that the exchange rates at this pH, calculated according to eqs. 1 and 2, yield very similar values, i.e. 191 s^-1 ^and 186 s^-1^, respectively.

### Possible mechanisms of exchange

**Figure 2:**
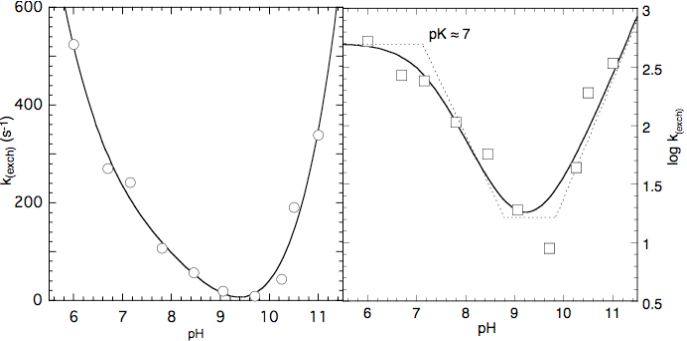
**pH dependence of the rate of N(5)-H exchange**. Conditions as detailed in the Legend of Fig. 1 and in the Experimental section. Panel (A): Semi logarithmic plot. The line through the data points was generated with a polynomial algorithm and has no specific meaning. Panel (B): Same data as in panel (A), however logarithmic representation of the exchange rate. The curve (—) through the data points was generated according to Dixon's criteria and using a pH independent rate = 10^2.7 ^(s^-1^), the pK_a _= 7.15 of reduced flavin (ionisation at N(1)-H), and apparent pK's = 8.7 and 9.8. The dashed lines (---) represent the single, unperturbed reactions that correspond to the processes described by equations 2–4 in Fig. 3. Their "breaks" occur at changes in ionisation state (pK_a_) or reflect changes in mechanism. See text for further details.

**Figure 3:**
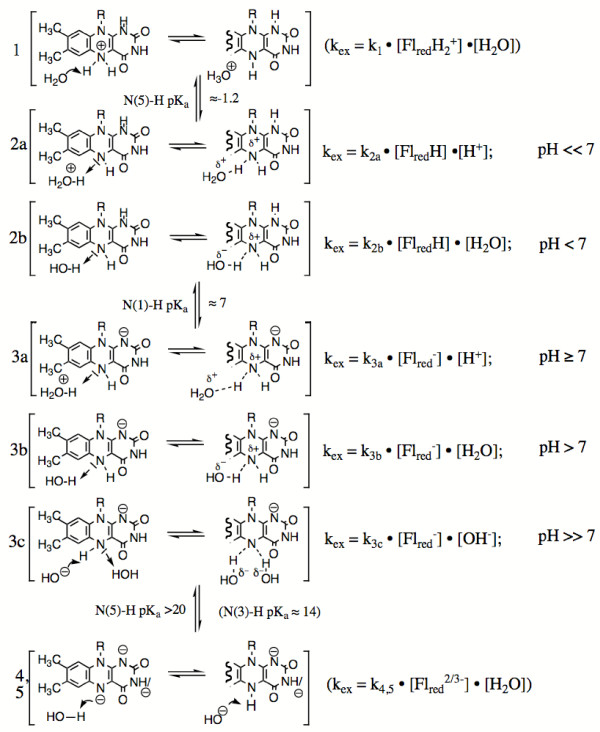
**Species involved in H-exchange at the reduced flavin position N(5) at its various stages of ionisation**. (1) represents the exchange of cationic N(5). Species (2) involves exchange of neutral reduced flavin with H^+ ^or H_2_O. (3) represent the modi of exchange of (mono-) anionic reduced flavin with H^+^, H_2_O or HO^-^. (4) and (5) are di- and tri-anionic reduced flavins that are likely to exchange with H_2_O.

The values obtained for the exchange rate in Tris buffer are summarized in Table 1 and are plotted versus pH in Fig. 2. The semi-logarithmic representation (panel A) shows that the exchange rate first decreases with increasing pH, it reaches a minimum around pH 9.5, this being followed by a steeper increase. The double-logarithmic representation (panel B) shows that the two segments have different profiles at low and high pH: The curve in this panel was generated based on the rules of Dixon [23] for the pH dependence of kinetic parameters of a species that is present in a pH dependent equilibrium linked by specific pK's and includes reactions with linear dependences from either H^+ ^or OH^-^, or no dependence. For the "Dixon analysis" several modi of exchange can be envisaged, 4 of which (2b, 3a, 3b, and 3c,) are relevant for the pH range investigated in this study (Fig. 3). These include the reduced flavin at its 4 possible ionisation states along with the structures involved. In the present case the species undergoing ionisation is reduced flavin with a pK_a _≈ 7 for the N(1)-H group [15]. At very low pH (Fig. 3, 1) the reduced flavin will be protonated at N(5) (pK ≈ -1.2, [24]) and exchange will be with H_2_O. This process (Fig. 3, 1) is not expected to play a role in the present case. Similarly, the [H_3_O^+^] dependent mechanism (2a) is probably not relevant since the data suggest that a corresponding slope is absent at pH <6. The neutral reduced flavin can exchange by reaction (2b) with H_2_O (Fig. 2B, horizontal segment up to pH ≈ 7). At pH 7–9 the [H_3_O^+^] dependent process (3a) then appears to become prominent (linear segment with slope = 1). The profile in Fig. 2B clearly indicates that at higher pH [OH^-^] dependent processes become dominant. This change in mechanism would be initiated by the switch of mode (2b) to the pH independent mechanism (3b)(short horizontal section between pH 9–10, Fig. 2B). This phase is envisaged to be followed by the [OH^-^] dependent mode (3c) (section at pH >10, linear increase with [OH^-^]). In Fig. 3, the equilibrium between species (2)  (3) that encompasses the structures included by the square brackets, reflects the pH dependent change in ionisation state of the reduced flavin with a pK_a _≈ 7. The point should be stressed that in the representation of Fig. 2B and according to the Dixon's rules only the "break" at ≈ pH 7 can be attributed to a microscopic pK. That corresponding to pH ≈ 8.7 is taken to reflect the change in mechanisms as outlined above, i.e. it is an apparent pK and has no structural significance since it cannot be attributed to ionisations of the exchange partners or of the buffer. The same probably holds true also for the break corresponding to a pH ≈ 10 since the pK_a _for the second ionisation of reduced flavin (N(3)-H) is estimated as ≈ 14.

**Table 1:** Line widths of the ^15^N resonance signal of the reduced FMN N(5) atom as function of pH and estimation of the N(5)-H pK_a_.

pH	Line width (Δν_1/2_-Δν_1/2_°) (Hz)	Exchange rate of N(5)-H (s-^1^)	pK_a _(calculated)
6	21	525	
6.7	42	270	
7.15	47	242	
7.8	34	107	
8.95	18	57	
9.05	6	19	
9.7	3	9	20.5
10.25	14	44	20.3
10.5	61	191	20.0
11	33.5	339	20.2

The flavin was 5 mM in 250 mM Tris + 100 mM NaCl. The estimation of the pK_a _value is based on the line width of the corresponding ^15^N signal as described in the text.

### Estimation of ionisation constants for the reduced flavin N(5)

The pK_a _of the flavin N(5)-H corresponding to the following exchange process:



can be estimated based on the general rates of proton transfer (k_1 _and k_-1_, as indicated in equation 3) between two exchanging species connected to the equilibrium constants K_flavin-N(5) _and K_H2O _as in equation 4:

Kflavin-N(5)=k1k−1⋅KH2O     (4)
 MathType@MTEF@5@5@+=feaafiart1ev1aaatCvAUfKttLearuWrP9MDH5MBPbIqV92AaeXatLxBI9gBaebbnrfifHhDYfgasaacH8akY=wiFfYdH8Gipec8Eeeu0xXdbba9frFj0=OqFfea0dXdd9vqai=hGuQ8kuc9pgc9s8qqaq=dirpe0xb9q8qiLsFr0=vr0=vr0dc8meaabaqaciGacaGaaeqabaqabeGadaaakeaacqqGlbWsdaWgaaWcbaGaeeOzayMaeeiBaWMaeeyyaeMaeeODayNaeeyAaKMaeeOBa4Maeeyla0IaeeOta4KaeeikaGIaeeynauJaeeykaKcabeaakiabg2da9maalaaabaGaee4AaS2aaSbaaSqaaiabigdaXaqabaaakeaacqqGRbWAdaWgaaWcbaGaeyOeI0IaeGymaedabeaaaaGccqGHflY1cqqGlbWsdaWgaaWcbaGaeeisaGKaeeOmaiJaee4ta8eabeaakiaaxMaacaWLjaWaaeWaaeaacqaI0aanaiaawIcacaGLPaaaaaa@4C60@

The exchange rate in the thermodynamically favorable direction can reasonably be assumed as being diffusion controlled (in this case k_-1 _= 10^10 ^M^-1^·s^-1^). For an estimation the HO^-^catalyzed process was selected since it is assumed to involve direct interaction with HO^- ^(3c, in Fig. 3) and appears to best approximate a linear dependence from pH. Thus the data between pH 9.7 and 11 were employed. The values summarized in Table 1 were obtained using the mentioned value for a diffusion controlled proton transfer rate and the equilibrium constant of water (= 10^-15.74 ^[25]). The average pK_a _value obtained from Table 1 is 20.2 (± 0.3).

**Figure 4:**
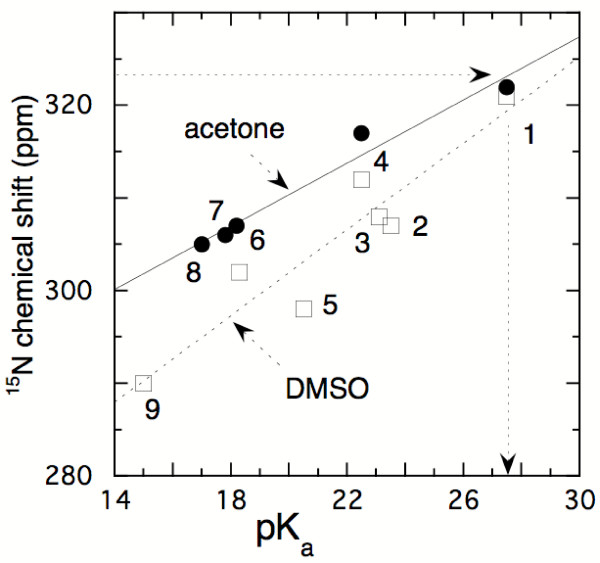
**Correlation of ^15^N-chemical shifts of aniline derivatives with their pK's and extrapolation of the pK_a _value for N(5)H of reduced FMN based on its ^15^N chemical shift**. The compounds are: (1) aniline, 2: 2-azaaniline; (3) 4-cyanoaniline; (4) 4-azaaniline; (5) 2,5-diazaaniline; (6) 4-nitroaniline; (7) 2-nitroaniline; (8) 4-chloro-2-nitroaniline; (9) 2,3-dinitroaniline (data from [34]). The horizontal arrow indicates the experimentally determined ^15^N chemical shift of N(5)H of reduced flavin and vertical arrow extrapolates to an estimated pK_a _26–30.

An estimation of the pK_a _of the flavin N(5)-H can also be derived from the chemical shift of the ^15^N(5) center. ^1^J^15 ^N-H coupling constants are mainly governed by the hybridization of the nitrogen atom. Since the N(5)-H coupling constant is similar to that reported for eneamines and aniline derivatives [22] it is reasonable to assume that the N(5) hybridization is similar also to that of a series of substituted anilines that are used in the correlation diagram of Fig. 4, where their chemical shift is plotted against their reported pK_a _values. From the chemical shift for the reduced flavin N(5) of ≈ 60 ppm [15] a pK >25 is obtained. While this value is higher than that derived from the exchange experiments it correlates with the chemical entity of a phenylene diamine substituted with a moderately electron-deficient (neutral) pyrimidine moiety. Inspection of the chemical structures in the lowest row in Fig. 3 shows that in a chemical system the species that deprotonates at position N(5)-H is a phenylene diamine linked to a pyrimidine carrying (already) two negative charges. The latter would undoubtedly increase the pK_a _for formation of the N(5) anion compared to a species in which neutral reduced flavin would ionize at position N(5)-H. The latter might be the case in a protein environment where specific charges or H-bridges might affect microscopic pK's. Nevertheless protein induced pK shifts with values >10 units are unlikely within the same molecule since it would require a stabilisation energy >59 KJ/Mol. A pK_a _value of ≥ 20 is also in good agreement with the estimation by Venkataram & Bruice (pK_a _= 19 – 23 [3]) deduced from the pH dependence of the rates of decay of reduced flavin model compounds that have the 4a,5-dihydro structure.

**Figure 5:**
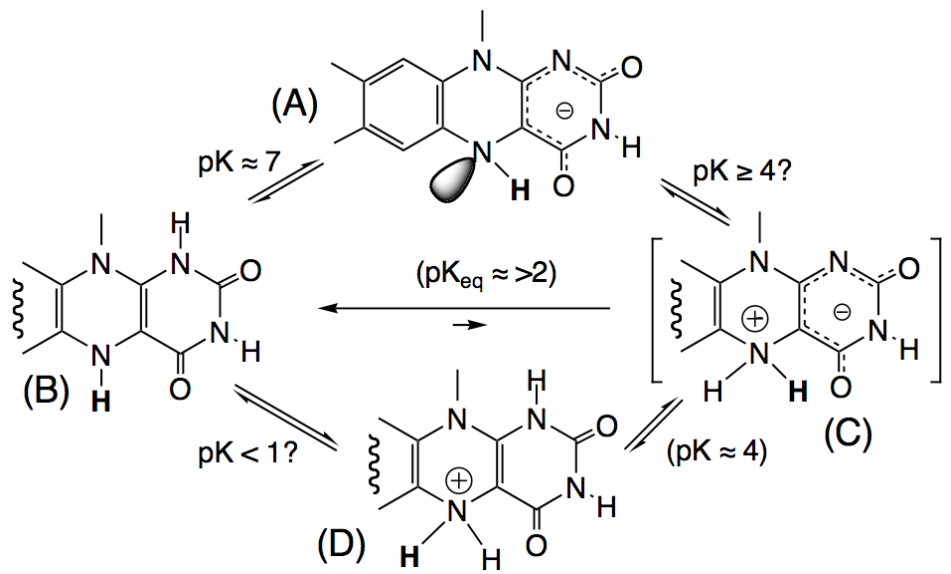
**Selected ionisations and tautomeric forms of reduced flavin**. (A) and (B) are the anionic, respectively the neutral forms of reduced flavin encountered at the active center of flavoproteins. The pK interconnecting (A) and (B) is ≈ 7 in the free state [26], but can vary strongly at the active center of proteins. Protonation of (A) at N(5) to yield (C) can not be observed in the free state but this chromophore is obtained by twofold alkylation at N(5) [26]. The pK's linking species (B), (C) and (D) have been estimated from the properties of appropriate model compounds [26]. See text for further details.

The second ionisation of the reduced flavin relevant in an enzymological context is the (de)protonation at position N(1) with a pK ≈ 7 (species (A)  (B), in Fig. 5). However, the locus of catalytic action is not N(1), but N(5). Thus, while the ionisation at N(1) plays a very important role in modulating the properties of reduced flavin and the flavin redox potential, it is unlikely to participate directly in base catalysis. A third ionisation that most likely is relevant in the biochemical context is the protonation of anionic reduced flavin (species (A) in Fig. 5) at position N(5)-H to yield the zwitterionic species (C). This species is not observed in free solution since the tautomeric equilibrium between species (B) and (C) favor the former by ≥ 2 orders of magnitude [26]. The N(5)-dialkylated analog to species (C), however, has been described [26] and is converted to (D) with a pK ≈ 4. The combination of the equilibria shown in Fig. 5 thus allow the estimation of the equilibrium between species (A) and (C) (Fig. 5) to correspond to a putative pK >4. While this "pK" has no direct significance in the free system it is easily conceivable that the protein could stabilize a negative charge at position N(1) as is probably the case with flavodoxins [15,27,28], thereby facilitating formation of a putative species (C). In this context it should be noted that the most nucleophilic functional group in (A) is not N(1)-C(2) = O, the locus of the negative charge, but N(5) as reflected by the main position of alkylation [26].

## Conclusion

Although a doublet for N(5)H of reduced free flavin can only be observed in the narrow pH range 8.5 – 10.5, it has been documented for reduced flavoproteins at pH values as low as 5 [15,27,28]. This suggests that in specific, reduced flavoproteins N(5)-H exchange is slow and that access of bulk solvent to this position is hindered. Occurrence of a doublet was observed in reduced thioredoxin reductase in which the N(1) atom is protonated [29]. Conversely, in reduced flavodoxins and many other flavoproteins the flavin N(1) position is not accessible for protonation [30] the flavin existing in the anionic form as long as the protein does not unfold (pH-dependent process). It thus appears that the rate of exchange at N(5)-H is not dependent on the ionisation state at N(1)-H but will be dictated by the environment and accessibility of solvent of the specific protein. However, no such conclusion can be drawn for the pH range 8.5 – 10.5 where the doublet of the N(5)-H is an inherent property of the reduced flavin. Unlike the N(5)H resonance in reduced free flavin the N(3)H resonance shows a narrow singlet over the whole pH range studied, indicating that proton exchange is fast. Therefore, the appearance of a doublet for this resonance in flavoproteins is compatible with hindered accessibility of this position for bulk water as previously interpreted [6].

The estimation of the pK_a _value of ≥ 20 for the N(5) group in reduced flavin might be an useful parameter for the formulation of mechanisms involving it. For instance Urban and Lederer [4,5] have postulated a deprotonated N(5) with a pK_a _of about 15 or even as low as 7 [5] as a catalytically relevant species in flavocytochrome b_2_. While our data cannot exclude such a pK_a_, it imposes specific energetic restraints for its formulation. On the other hand the reduced flavin N(5)-H with an estimated pK_b_, ≥ 4 for the free molecule possesses sufficient basicity to act as a base according to mechanism 3b (Fig. 3). Clearly, this property can be modulated by the protein environment to suit specific purposes. The conclusion is thus that the reduced flavin N(5)-H has properties that qualify it as a base catalyst for biochemical reactions. This could be realized in several cases: The recent elucidation of the structure of chorismate synthase [12,13] suggests that N(5)-H of the reduced flavin cofactor is involved in the abstraction of the hydrogen in the C(6) *pro-R *position of the substrate 5-enolpyruvylshikimate 3-phosphate. A similar mechanism, abstraction of a proton from the α-CH_2 _(*pro-R*) of an amine by monoamine oxidase [14], seems to be emerging, replacing the previously proposed single electron transfer mechanism. Likewise, N(5) appears to be involved in the elimination of halide from β-Cl-alanine catalyzed by D-amino acid oxidase [9]. At the active sites of these enzymes there is no amino acid functional group that could participate in the required base catalysis. The data presented in this paper thus sustain the notion that N(5) of the reduced flavin cofactor can play a direct role in the catalysis of some flavin-dependent enzymes, an involvement that has not yet been addressed in sufficient detail. Our studies towards a characterization of the hydrogen exchange processes at this position therefore lay the foundation for a critical assessment of this role.

## Methods

### Experimental details

The synthesis and purification of [1,3,5,10-^15 ^N_4_]-7-methy1-10- ribitylisoalloxazine-5'-phosphate was described previously [31,32]. ^15^N-NMR measurements were performed at 15°C, if not otherwise stated, with a Bruker AM 500 NMR instrument equipped with an Aspect 3000 and a temperature control unit. NMR-spectra were recorded with 12 μs pulses (= 30° flip angle) and a relaxation delay of 2 sec. In a typical experiment 10 mm Wilmad precision NMR tubes contained 1.8 ml of a 3 to 8 mM solution of flavin (or otherwise indicated in the text) in 250 mM Tris/100 mM NaCl-buffer and 0.2 ml D_2_O for field frequency lock. Reduction of the flavin solution was achieved by flushing the sealed NMR tube with argon before a two to three fold excess of a concentrated dithionite solution was added with a syringe. The pH of the resulting reduced solution was measured after the NMR experiment with a pH meter from Radiometer (Copenhagen, Sweden) equipped with a glass electrode from Ingold (Frankfurt, Germany). All ^15^N chemical shifts are expressed relative to liquid ammonia at 25°C and are corrected for bulk volume susceptibility. Neat CH_3 _^15^NO_3 _(™(CH_3_NO_3_) - ™(NH_3_) = 381.9 ppm) was used as an external standard according to Witanowski et al [33]. The proton exchange rate (1/τ_e_) was calculated according to Gutowsky et al [34] in the case of a fast exchange reaction, and according to Grunwald et al [35] in the case of a slow exchange reaction.

## Authors' contributions

PM carried out most NMR experimental work with CS and evaluated the NMR data, he participated in drafting the manuscript.

SG conceived the study, participated in its design and coordination, as well as in the evaluation of the kinetic data and drafting of the manuscript.

FM synthesized the labeled flavins used in the cooperative studies with the group of HR, and was involved in the drafting and finalization of the paper.
